# Recommendations for the FAIRification of genomic track metadata

**DOI:** 10.12688/f1000research.28449.1

**Published:** 2021-04-01

**Authors:** Sveinung Gundersen, Sanjay Boddu, Salvador Capella-Gutierrez, Finn Drabløs, José M. Fernández, Radmila Kompova, Kieron Taylor, Dmytro Titov, Daniel Zerbino, Eivind Hovig

**Affiliations:** 1 Center for Bioinformatics, University of Oslo (UiO), Oslo, Norway; 2 European Molecular Biology Laboratory, European Bioinformatics Institute, Hinxton, UK; 3 Life Sciences Department, Barcelona Supercomputing Center (BSC), Barcelona, Spain; 4 Department of Clinical and Molecular Medicine, NTNU – Norwegian University of Science and Technology, Trondheim, Norway; 5 Department of Tumor Biology, Institute for Cancer Research, Oslo University Hospital (OUH), Oslo, Norway

**Keywords:** FAIR, functional genomics, epigenomics, genomics, metadata, interoperability, genomic tracks, sequence annotations

## Abstract

**Background: **Many types of data from genomic analyses can be represented as genomic tracks, *i.e.* features linked to the genomic coordinates of a reference genome. Examples of such data are epigenetic DNA methylation data, ChIP-seq peaks, germline or somatic DNA variants, as well as RNA-seq expression levels. Researchers often face difficulties in locating, accessing and combining relevant tracks from external sources, as well as locating the raw data, reducing the value of the generated information.

**Description of work: **We propose to advance the application of FAIR data principles (Findable, Accessible, Interoperable, and Reusable) to produce searchable metadata for genomic tracks. Findability and Accessibility of metadata can then be ensured by a track search service that integrates globally identifiable metadata from various track hubs in the Track Hub Registry and other relevant repositories. Interoperability and Reusability need to be ensured by the specification and implementation of a basic set of recommendations for metadata. We have tested this concept by developing such a specification in a JSON Schema, called FAIRtracks, and have integrated it into a novel track search service, called TrackFind. We demonstrate practical usage by importing datasets through TrackFind into existing examples of relevant analytical tools for genomic tracks: EPICO and the GSuite HyperBrowser.

**Conclusion: **We here provide a first iteration of a draft standard for genomic track metadata, as well as the accompanying software ecosystem. It can easily be adapted or extended to future needs of the research community regarding data, methods and tools, balancing the requirements of both data submitters and analytical end-users.

## Introduction

Genomic track files were originally designed and optimised to be displayed within genomic web browsers, but have gradually become a *de facto* standard to store, distribute and analyse genome-wide datasets, mainly because of their efficient compression and indexing utilities. Many bioinformatics analyses are now being distributed, either privately or publicly, using such files. They are still predominantly used for graphical display, but can also be queried by statistical analysis tools, such as the GSuite HyperBrowser ^1^, EPICO ^2^, DeepBlue ^3^ or the IHEC DataPortal ^4^.

File formats used to represent tracks were not designed with FAIR data principles ^5^ in mind, in particular with respect to metadata. Thus, their potential impact through re-use is greatly limited. Without proper metadata, an understanding of the protocol through which a track file was generated requires intensive literature curation and/or personal communication with the data generators, and these approaches are hampering usage. Further, if the exact provenance of a track file is not well understood, any downstream re-analysis is prey to artefacts. Thus, poorly annotated track files are rendered virtually useless for automated re-use.

Furthermore, no central repository exists dedicated to storing track files and curated metadata. Instead, the track repositories are divided by species (mainly human vs. other systems) and domain ( *e.g.,* epigenomics, cancer, common variants, or rare-disease variants), with data portals typically being created during the life cycle of larger consortium undertakings ( *e.g*., ENCODE or ICGC). The Track Hub Registry is a global centralised collection of public track hubs, and includes the metadata for a diverse collection of genomic tracks. However, the metadata content in the Track Hub Registry is not curated, and search facets are limited to high-level trackhub attributes.

### Motivation

Significant investments have gone into the generation of genomic tracks both within large consortia and independent groups, but the metadata is fragmented and disparate. Currently, significant legwork is required in order to identify, collect and consolidate a set of tracks to be used for a given research project. This is due to the often low quality of metadata annotations, like erroneous and missing metadata, duplicate attributes and/or records. More systematic deficiencies include difficulties accessing metadata and data (using various APIs and *ad hoc* scripts), and even a lack of adherence to the established formats for data and metadata. There is also a lack of standard metadata attributes across repositories and/or data types for simple filtering tasks. Critical information in need of standardization include the cell or tissue type of the sample, the assay type and main target of the experiment (if any), information on methods used to generate the track, as well as properties of the track files themselves such as the genome assembly version used ^6^, the level and type of data condensation, and the geometric properties of the track ^7^. Further, in many settings, manual interpretation of the metadata may be demanding, as non-standard terms are used, causing significant confusion and uncertainty, which results in a lack of trust in the data.

With the use of sufficient standards for metadata, and with matching development of functionality to make genomic tracks reusable, it is conceivable that one could mobilize all the available datasets world-wide relevant to a research question. This would positively impact both cost-efficiency by reuse of existing data as well as contribute to the evaluation of available datasets prior to the design of new projects. Ideally, all useful data should be easily searchable across repositories, downloadable, interoperable in terms of widely accepted formats, and directly usable by research software. Another formulation of this concept would be through the FAIR terms ^5^: Findable, *e.g.,* implementing metadata standards across track repositories; Accessible, *e.g.,* making use of existing open protocols for accessing both data and metadata, even in the light of controlled access; Interoperable, *e.g.,* utilizing open, well-defined and usable formats and protocols for track data; and Reusable, *e.g.*, by describing data with sufficient attributes, such as provenance and usage policy, for reuse through a well-integrated ecosystem of functions across different fields of research.

Based on our work in the ELIXIR Implementation Study on FAIRification of genomic tracks, we describe our efforts towards enabling genomic track FAIRness, through a description of a draft genomic track metadata standard, the development of a search function, and a demonstration of how interoperability and reuse can be achieved for the end-user by its inclusion in larger track registries.

### Existing standards

Domain-specific metadata standards exist that guide current efforts to annotate genomic datasets (see Table 1 for a summary of object types across these data models and their required attributes). The “International Nucleotide Sequence Database Collaboration” (INSDC) deposition model was developed jointly by NCBI, EMBL-EBI and DDBJ to facilitate genomic data exchange ^i, ii^, and serves as a model for many of the standards described below. In particular, each data file is assigned to an “Experiment”, which links out to a specific “Study” and a specific “Sample”.

**Table 1.** Summary of required attributes for metadata standards related to genomic track files.

Standard	Object	Required attributes	Allowed values
FAANG	Analysis	Input data	String
FAANG	Analysis	Reference data	String
FAANG	Analysis	Analysis protocol	String
FAANG	Analysis	Total reads	Number
FAANG	Analysis	Mapped reads	Number
FAANG	Experiment	sample	BioSampleID
FAANG	Experiment	assay type	Enum ('ChIP-Seq', 'RNA-Seq of coding RNA'...)
FAANG	Experiment	sample storage processing	Enum ('Fresh', 'Formalin fixed'...)
FAANG	Experiment	sampling to preparation interval	String with number + unit
FAANG	Experiment	extraction protocol	String
FAANG	Sample	SampleName	String
FAANG	Sample	Material	Enum ('Cell line', 'Organism'...)
FAANG	Sample	project	"FAANG"
HyperBrowser	AnalysisFile	URI	URI
HyperBrowser	AnalysisFile	Genome build	String (UCSC assembly versions)
HyperBrowser	AnalysisFile	File suffix	String
HyperBrowser	AnalysisFile	Data type/represented concept ( *e.g.*, narrow peaks, signal, single mutations...)	Enum
HyperBrowser	AnalysisFile	Target (main target of dataset, *e.g.*, antibody/ disease/gene/... To be used across technologies)	String
HyperBrowser	AnalysisFile	Cell type	String
HyperBrowser	AnalysisFile	Tissue type	String
HyperBrowser	AnalysisFile	Experiment type	String
IHEC	Experiment	EXPERIMENT_TYPE	Enum ("DNAme", "RNA-Seq"...)
IHEC	Experiment	EXPERIMENT_ONTOLOGY_URI	OBI
IHEC	Experiment	LIBRARY_STRATEGY	Enum ('RNA-Seq', 'ChIP-Seq' ...)
IHEC	Experiment	MOLECULE_ONTOLOGY_URI	SO
IHEC	Experiment	MOLECULE	Enum ('Total RNA', 'Genomic DNA', ...)
IHEC	Sample	SAMPLE_ONTOLOGY_URI	EFO, CL or UBERON depending on type
IHEC	Sample	DISEASE_ONTOLOGY_URI	NCImetathesaurus
IHEC	Sample	DISEASE	String
IHEC	Sample	BIOMATERIAL_PROVIDER	String
IHEC	Sample	BIOMATERIAL_TYPE	Enum ("Cell Line", "Primary tissue"...)
INSDC	AnalysisFile	filename	string
INSDC	AnalysisFile	filetype	Enum
INSDC	AnalysisFile	checksum_method	Enum
INSDC	AnalysisFile	checksum	string
ISA-tab	Assay	Measurement Type	Ontology Annotation
ISA-tab	Assay	Technology Type	Ontology Annotation
ISA-tab	Assay	Technology Platform	String
ISA-tab	Investigation	Identifier	String
ISA-tab	Investigation	Identifier	String
ISA-tab	Investigation	Title	String
ISA-tab	Investigation	Description	String
ISA-tab	Investigation	Submission Date	Representation of a ISO8601 date
ISA-tab	Investigation	Public Release Date	Representation of a ISO8601 date
ISA-tab	Investigation	Publications	A list of Publication
ISA-tab	Investigation	Contacts	A list of Contact
ISA-tab	Study	Identifier	String
ISA-tab	Study	Title	String
ISA-tab	Study	Description	String
ISA-tab	Study	Submission Date	Representation of a ISO8601 date
ISA-tab	Study	Public Release Date	Representation of a ISO8601 date
ISA-tab	Study	Publications	A list of Publication
ISA-tab	Study	Contacts	A list of Contact
ISA-tab	Study	Design Type	Ontology Annotation
ISA-tab	Study	Factor Name	String
ISA-tab	Study	Factor Type	Ontology Annotation
Track Hub	Analysis	Contact e-mail address	String
Track Hub	AnalysisFile	An assembly identifier	UCSC nomenclature
Track Hub	AnalysisFile	A filetype	Enum
Track Hub	AnalysisFile	A URL	String
Track Hub	AnalysisFile	A short label	String
Track Hub	AnalysisFile	A long label	String
Zenbu	AnalysisFile	FileFormat	String
Zenbu	AnalysisFile	Date	String
Zenbu	AnalysisFile	ProtocolREF	String
Zenbu	AnalysisFile	ColumnVariable (string descriptions of each column)	String
Zenbu	AnalysisFile	ContactName	String
Zenbu	AnalysisFile	ContactEmail	String

A number of international efforts exist to extend the INSDC submission model to specific domains and/or organisms. For instance, IHEC ^8^ is an umbrella organisation that brings together large epigenomic data production efforts, such as BLUEPRINT ^9^, ENCODE ^10^, and other large-scale initiatives. Each separate initiative produces tracks and deposits their data separately. However, IHEC brings together their metadata to a central location, so as to facilitate integrative analysis. Its data model is an extension of the INSDC submission model, to which attributes have been added, and where specific ontologies are recommended ^iiii^.

“Functional annotation of animal genomes” (FAANG) ^iv^ was created to coordinate the collection of functional genomics data (in particular epigenomics) across animals (in particular livestock). Its data model ^v^ is also an extension of the INSDC model, also with recommended attributes and ontologies.

ICGC ^vi^ gathered the data from over 90 different cancer genome projects, as well as their heterogeneous analyses. The Data Coordination Center defined a set of data models ^vii^, which is able to hold both anonymized metadata of the patients and samples, metadata of the experiments and analyses, as well as the results. It also established a federated cancer database, where the different partners are responsible for pushing their patients, samples, experiments and analysis metadata and data, having been translated and normalized to the ICGC data models. It became the seed for the more complete initiative of “Pan Cancer Analysis of Whole Genomes” (PCAWG) ^viii^.

In addition, the Genomic Data Commons (GDC) ^ix^ is a research program of the National Cancer Institute (NCI) to provide the cancer research community with a unified data repository that enables data sharing across cancer genomic studies in support of precision medicine. Data and metadata are submitted to the GDC in standard data types and file formats through the GDC Data Submission Pipeline. The GDC hosts and distributes previously generated data from The Cancer Genome Atlas (TCGA), Therapeutically Applicable Research to Generate Effective Treatments (TARGET), and other programs. The GDC data model is based off the DAta Tags Suite (DATS) ^11^, a general metadata model for biological results, which itself was designed to mirror the Journal Article Tag Suite (JATS) ^x^, required for submission into PubMed ^xi^ to index publications.

Finally, the ISA framework ^xii^ provides metadata standards for annotating experimental datasets, with detailed metadata configurations designed by expert groups for most common experiment types and domains, represented in ISA-TAB or ISA-JSON formats. The ISA data model is built around some core metadata categories: Investigation, Study, and Assay. In addition, the ISA framework provides tools for annotation, curation, or conversion of metadata, and also deployment tools that follow the requirements of public repositories, such as ArrayExpress ^xiii^ and the European Nucleotide Archive (ENA) ^xiv^, as well as selected journals. Noteworthy, the ISA framework has recently been selected as one of the Recommended Interoperability Resources ^xv^ by ELIXIR.

Other organizations are important as mechanisms to promote the adoption of standards by the international research community and include, for instance, the Global Alliance for Genomics and Health (GA4GH) ^xvi^. This is an organization focused on the creation of policy frameworks and technical standards, which allow sharing of genomic and medical data ^xvii^ in a responsible ^xviii^ way. While much of the data types covered by GA4GH are not genomic tracks, some of the datasets can be displayed as genomic tracks, for example genotype data. Of relevance is also the refget API ^xix^, a GA4GH-approved standard that provides unambiguous access to reference sequences from unique checksum identifiers based on the sequence content itself.

### Existing genomic track metadata consumers

In practice, standards are often defined and implemented by widely used tools, who set *de facto* standards on what is a valid input or not. We therefore list below some main consumers of track hub files.

The Track Hub Registry ^xx^ serves as a common entry point to register data collections into Ensembl ^12^ and UCSC ^13^ genome browsers. Its exchange format is a set of text files, collectively referred to as a track hub ^xxi^. In effect, each genomic track is necessarily assigned the information required to display it. In particular, this includes its URL, the genome it maps to and free text descriptions. Optional attributes can then be attached to each track, in particular display settings, but also experimental metadata. Track metadata can be inserted into the track hub, either a) directly in the track hub file, as a list of key/value pairs, b) as an ancillary TSV file, or c) as an ancillary TagStorm file ^xxii^.

On August 1st 2018, we surveyed the content of the Track Hub Registry. There were 4,294 track hubs spread across 10 species that all combined accounted for a total of 103,301 genomic tracks. Sampling through the BLUEPRINT track hub shows that each track contains on average 1.8GB of data. Because Ensembl have fully adopted track hubs and produce them automatically, most of the track hubs map to plant species ( *Arabidopsis thaliana, Zea mays, Oryza sativa Japonica Group, Glycine max, Sorghum bicolor, Triticum aestivum, Solanum lycopersicum, Brachypodium distachyon, Brassica napus*), while only 3% map to human. However, the average human track hub has far more tracks (mean 218 tracks per hub) than the average plant track hub (mean 18 tracks per hub). A survey of metadata keys available on the track hubs suggests a great disparity in usage as provided in Table 1.

The Zenbu browser ^xxiii^ was initially developed to distribute the data produced by the FANTOM consortium. Data can be uploaded to it via its OSCTable format ^xxiv^, a general tab-delimited file format that subsumes the BED, GFF and SAM files used by genomic browsers. One distinction is that the format allows the writer to explicitly define data types and experiments (string identifiers). In addition, the file header allows the writer to optionally insert metadata describing the file or the experiment identifiers defined in the column names.

The Genomic HyperBrowser ^14^ is a web-based framework aiming to provide a complete downstream solution for statistical analysis of track data, starting after data processing steps like ChIP-seq peak calling or variant calling has taken place. The implemented analyses are based on rigorous statistical fundamentals and methodologies, and include colocalization analyses, but also analyses making use of signal values and 3D DNA structure, as well as methods for clustering and visualisation.

The latest expansion, GSuite HyperBrowser ^1^, constitutes a redesign of the analysis workflow with a focus on defining and managing collections of related tracks with associated metadata throughout all analysis steps. With the GSuite expansion, tools were created for searching and downloading data from established track repositories, such as Roadmap Epigenomics or ENCODE. This work laid bare a range of issues with the then current state of track metadata, starting a process that has led to the realisation of this implementation study.

Several efforts have been carried out to collect and organise track data, presented for use as reference data for specific analysis methodologies, including EpiExplorer ^15^, ColoWeb ^16^, GenomeRunner ^17^, LOLAweb ^18^, and epiCOLOC ^19^. Typically, the user provides a query track as input, which is then compared with the available reference datasets in order to generate relevant associations. Overall, such efforts are limited to organize tracks based on a few metadata fields, such as project, tissue type, assay type and assay target. Also, such track collections are often collected and organized prior to publication, but they are seldom updated afterwards. Similar efforts in the future would benefit significantly by the FAIRification of track metadata suggested here, as would other tools and services providing downstream analysis of track data, including machine learning methods ^20– 22^.

## Metadata recommendations

**Figure 1.**
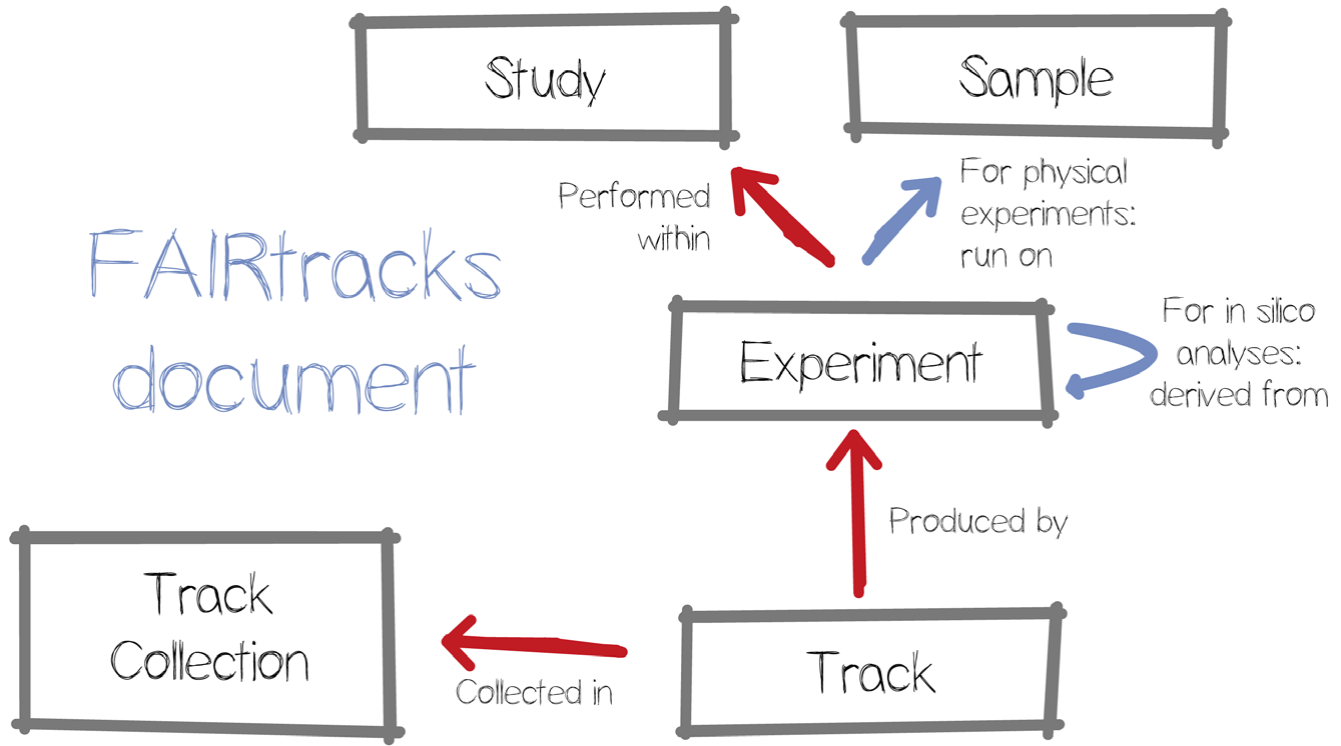
Overview of the key objects in the proposed data model, and the relationships between them.

Building upon prior art, we started by creating a data model around four key objects: studies, samples, experiments, and tracks (see Figure 1). The atomic data element is the Track, a genomic data file, generally in a binarized and indexed file format, such as BigBed ^xxv^, BigWig ^xxvi^ or BCF ^xxvii^, optimised for display in a genome browser. Each track is generated by an Experiment, whether physical or *in silico*. Physical experiments can be mapped for example to experiments in the European Genome-phenome Archive (EGA) ^23^, although to our knowledge, *in silico* experiments do not have an authoritative identifier system. Physical experiments link out to Samples, as described in BioSamples ^24^. Next, a set of experiments are contained within a study, which is akin to the EGA Study objects. Finally, one or more tracks are grouped into a Track Collection, which is the outer scope of the model and directly matches the existing track hub object. A Track Collection can also refer to an *ad hoc* collection of tracks, *e.g.*, documenting the input data of published analyses.

We then defined the required and optional attributes for each object type ( Table 2). The first consideration when defining a data model is to strike a compromise between the work imposed on the producer and the consumer of the metadata. The file must be clear and useful, yet not be such of a burden to compile that submitters would circumvent metadata entry. We therefore opted to only require attributes that appeared necessary for generic re-analysis of the data, while at the same time promoting FAIRness, by inclusion of resolvable references to relevant existing metadata records in external resources. To ensure practical usability, we generated a large test metadata record to test our proposal for ambiguities and difficulties, as described in more detail below. This testing phase clarified a need for an automated intermediate step between metadata curation and consumption, which in most cases simply meant to augment resolvable identifiers with human readable versions of the same. Adding this augmentation step simplified metadata entry, while simultaneously strengthened metadata search and extraction, in both automated and manual usage scenarios.

**Table 2.** Key attributes of FAIRtracks objects.

**FAIRtracks document**	Version id, version date, ontology versions, URL to original source
**Track Collection**	Name, description, URL to original source, contact info
**Study**	Name, publications, contact info
**Sample**	Species, biospecimen class, sample type ( *e.g.*, specific cell type, cell line, tissue), phenotype
**Experiment**	(Sample OR upstream experiment), technique, biological target ( *e.g.*, gene, motif, phenotype), lab/ compute protocols
**Track**	Assembly details, file URL, label, description, ID of source collection, IDs of raw files, file format, type of condensed data, genometric track type, checksum

One major design consideration was allowing for multiplicity in the relations between these types of objects. For example, a sample can be associated with multiple experiments and an experiment with multiple track files ( *e.g.*, different analysis results), but, conversely, a track file can be linked with multiple samples ( *e.g*., an aggregated statistic). Given these constraints, a hierarchical model was not possible, so we opted to define these objects independently, and then link them up by reference.

The schema was finally formalised as a JSON Schema, as this generic technology is widely used, and benefits from well-maintained parsers and validators in an array of programming languages. To a large extent, we re-used existing FAIR resources, such as ontologies or identification services that can be referenced with a CURIE and resolved via the Identifiers.org ^25^ service, which maps a CURIE to the Internet locations where the referenced item is hosted.

## Prototype implementation

Having defined a theoretical standard, we tested its feasibility and usability by generating a test object that fits this specification. To ensure robust testing of our framework, we chose one of the largest existing track hubs, namely the BLUEPRINT track hub, formatted its metadata according to our proposed requirements, and then propagated it via representative services in the genomic track ecosystem ^xxviii^:

- The Track Hub Registry ^xxix^ allows data producers to register and share trackhub files. These files are then parsed and indexed by the Ensembl and UCSC genomic browsers and are thus available for searching on both of these widely used services. Currently, the main requirement for submission into the Track Hub Registry is to provide a correctly formatted track hub file that can be displayed on the genomic browser. Because its purpose is to facilitate the sharing and distribution of genomic analysis results, we do not plan to alter these requirements. Rather, datasets that conform to the metadata standard presented here would be highlighted and made easily available for transfer to downstream tools. To facilitate such transmission, the Track Hub Registry’s API now allows remote querying of its content.
- The FAIRtracks validation server ^xxx^ provides a RESTful API to allow FAIRtracks standard adopters to check whether the track metadata correctly adheres to the FAIRtracks JSON Schema. The server is based on standard JSON Schema validation technology, extended with additional Python modules that allow powerful local checks, such as validating ontology terms against specific ontology versions, or checking CURIEs against the registered ones at Identifiers.org. The extended validator also supports document-set restrictions, like *unique* constraints enforcement and *foreignProperty* checks. The JSON Schema validator extensions are being implemented also in Java.
- The FAIRtracks augmentation service ^xxxi^ is a RESTful API that takes as input a FAIRtracks-annotated JSON document containing the minimally required fields and automatically generates an extra set of “augmented” fields, containing human-readable ontology labels, ontology versions, and otherwise useful content for downstream users.
- TrackFind ^xxxii^ is a search and curation engine for FAIR genomic tracks. It supports crawling of the Track Hub Registry and other data portals to fetch track metadata. Crawled metadata can be accessed through hierarchical browsing or by search queries, both through a web-based user interface, and as a RESTful API. TrackFind supports advanced SQL-based search queries that can be easily built in the user interface, and the search results can be browsed and exported in JSON or GSuite format ^xxxiii^. The RESTful API allows downstream tools and scripts to easily integrate a TrackFind search, currently demonstrated by the GSuite HyperBrowser and EPICO. We plan to extract the curation functionality from TrackFind into a separate toolset, and extend it according to user needs.
- JSON-to-GSuite ^xxx^ is a RESTful service for converting FAIRtracks-annotated JSON documents into the GSuite format ^xxxiv^. This conversion is needed primarily to enable the TrackFind client in the GSuite HyperBrowser to output track metadata in the GSuite format, in order for the track collections that results from particular search queries to be consumable by existing manipulation and analysis tools in the framework.
- The EPICO Data Analysis Portal is a generalization from the BLUEPRINT Data Analysis Portal ^xxxv^. It is designed following the client-server paradigm, which also supports having different pluggable data backends at its REST API. The integration with the FAIRtracks ecosystem ^xxxvi^ is realised through a new ‘fairtracks’ backend, which translates queries and the data model to both the Track Hub API and to remote genomic tracks. Also, the REST API and the underlying EPICO data model has been generalized to deal with different reference genomic assemblies and organisms. The EPICO web frontend is also being updated, first to support dealing with different organisms and multiple reference genomic assemblies. Lastly, additional views are in preparation, in order to provide insightful views for the different kinds of genomic tracks, based on the various kinds of analyses and experiments to be undertaken.
- The GSuite HyperBrowser ^xxxvii^ has been extended with a webtool that allows for querying FAIRtracks-annotated metadata available through TrackFind. This TrackFind client tool creates a GSuite file containing metadata for the resulting tracks, including remote URLs to the relevant track files. Existing tools in the framework can further download the track data to the server and prepare them for further analysis using the statistical analysis tools included with the GSuite HyperBrowser extension ^1^. As a proof-of-concept, the BLUEPRINT metadata were queried and used in a demonstration analysis ^xxxviii^ of Multiple Sclerosis-associated DNA GWAS variants vs. DNAse I hypersensitivity sites from 58 BLUEPRINT normal cell type samples.

## Discussion

**Figure 2.**
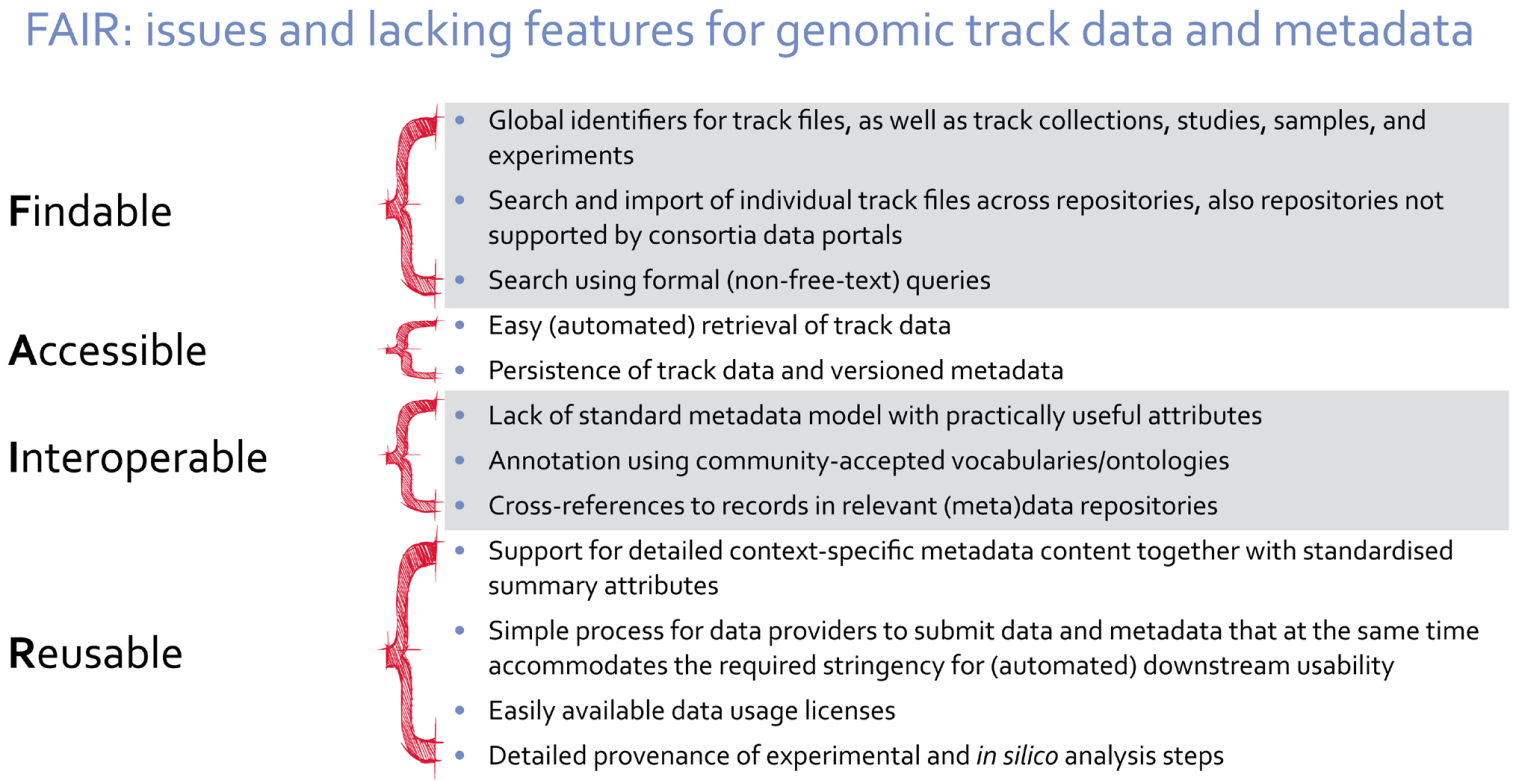
Important topics where the current state of track data and metadata have potential for improvements, as mapped to the FAIR ^5^ recommendations.

We have presented a summary of existing metadata standards. We think there is an unmet need ( Figure 2) for a standard for track metadata, together with a related infrastructure, aimed primarily at simplifying the day-to-day activities of researchers integrating genomic track data of varying types and from different sources, whether this is by manual web access, by *ad hoc* scripting, by the use of track analysis tools, or by the implementation of novel methodologies as new tools. Of great importance in such scenarios is how well a solution improves on the relatively poor state of accordance to the FAIR recommendations ^5^ with current track metadata. The following discussion of our FAIRtracks draft standard and the related proof-of-concept infrastructure is thus organized according to the FAIR principles.

### Findable

*F1. (Meta)data are assigned a globally unique and persistent identifier*

There are few solutions for assigning globally unique identifiers to track files themselves. In many cases, only the raw sequence files are assigned identifiers, typically accession number to core data repositories, such as the Sequence Read Archive (SRA) ^26^ or the European Genome-Phenome Archive (EGA) ^23^. The ENCODE ^10^ project represents a welcome exception, as they have assigned Identifiers.org-resolvable identifiers to each single track file with associated web-pages.

Track files typically contain condensed data returned from bioinformatics tools, which are run as part of defined workflows. As such, the track files are prone to contain biases due to specific parameter settings or workflow states, warranting the common advice to researchers to redo all analyses from the sequence files, with full control of the complete process. However, this might be impractical or unfeasible. Furthermore, track analysis is often exploratory in nature and a certain amount of error is often acceptable. The ability to uniquely identify specific track files is thus crucial for track analysis with positive consequences for reproducibility ^27^.

A globally accessible service to assign and register identifiers to single track files and collections of tracks is currently missing. We strongly recommend the implementation of a track registry that also preserves the full context surrounding the track files, with global identifiers to not only the track files, but also to the associated metadata. Our FAIRtracks draft standard could be advantageous for use as a basis for such a registry of track metadata. For now, we are leveraging the widespread adoption of the document identifier (DOI) by requiring a FAIRtracks document to be published and identified with a DOI. We require the publisher to support DOI versioning and also the possibility of reserving a DOI prior to publication (to include the DOI in the published file itself). We currently recommend using Zenodo ^xxxix^ as the publishing platform, as the service supports both features, but other platforms are also possible as long as both DOI versioning and reservation are possible. FAIRtracks is easily extendable to support other global identifier types.

To improve the provenance of analyses making use of genomic track data, *e.g.*, as part of a publication, it would be useful to also be able to uniquely identify specific *ad hoc* collections of tracks. One way to support this would be to only allow a single track collection in a FAIRtracks document, and then refer to the DOI. There is, however, an issue with granularity, as the number of published datasets in that case would be impractically high, as no publishing platform supports identifiers at a sub-dataset level in combination with versioning and pre-reservation of DOIs. Creating a separate service to assign and register identifiers at the track collection level seems a better solution (see A2 below).

*F2. Data are described with rich metadata*

This FAIR principle (and also R1) concerns the importance of providing generous metadata, and to not limit their availability due to assumptions about the users. This principle seems to stand in direct opposition to our goal of proposing a minimal standard for track metadata, based upon, precisely, our ideas of usability. However, the FAIR principles relate to the metadata content, and not its structure. FAIRtracks explicitly allows for any metadata property to be added by document creators. Indeed, the schema is designed so that existing repositories with curated track metadata can be converted in batch to follow FAIRtracks, retaining all the non-conforming metadata fields. Rather than being a schema for metadata completeness, FAIRtracks also advocates the inclusion of machine-resolvable identifiers that refer to external metadata records deployed in external community-standard repositories, such as BioSamples ^24^.

*F3. Metadata clearly and explicitly include the identifier of the data they describe*

As a global track repository currently does not exist, we were not able to require the inclusion of global identifiers to track data files. FAIRtracks does, however, require the inclusion of URLs to track files, but this is without any guarantees of persistence or uniqueness. Even if a track file does not have a direct identifier attached, one can often instead include an identifier to a parent record, *e.g.,* to an experiment or a study. As FAIRtracks requires a global identifier for the metadata document itself (using DOI), it should be possible to uniquely identify a track file from a joint identifier containing the DOI of a FAIRtracks document and the local identifier for a track file within that document.

One of the main goals of this study has been to make it easier for researchers to make use of historical track sources. Unfortunately, many existing track sources contain little metadata and seldom identifiers referring to external records of, *e.g.*, samples or studies, and also no domain standard for which external repositories to refer to. We thus concluded that we at least for now would recommend, but not require, the inclusion of global identifiers to external records for the main object types.

*F4. (Meta)data are registered or indexed in a searchable resource*

In parallel to the specification of the FAIRtracks draft standard, we have implemented a central search service named TrackFind. Our experience from the development and usage of track analysis software has clearly shown that the most useful form of track search is a formal search with a predefined set of values for each attribute, in contrast to a free-text search interface which is a typical choice for integrative services. The usefulness of such categorical metadata can be witnessed in the design of most track data portals. For instance, in the IHEC Data Portal ^xl^, the datasets are even visualized as a matrix defined by categories of epigenomes ( *e.g.,* cell or tissue types) in one axis, and assay targets ( *e.g.,* transcription factors or histone modifications) in the other. The main challenge in order to make such a search service work across categorical metadata from a range of sources is in harmonizing the metadata.

We have attempted to harmonize metadata at different levels: 1. We have selected and named a set of core attributes. 2. We have limited the possible values for these attributes to those contained in certain ontologies or limited vocabularies. 3. We have tried to balance the various constraints posed on the metadata structure from, *e.g.*, specific types of track data, experimental techniques, biospecimen types, or ontology richness, with the need for uniform metadata attributes and categories across heterogeneous track sources and data types. With FAIRtracks, we believe we have managed to balance the various constraints in a manner that makes the schema uniquely suitable for a unified formal search service. With TrackFind, we provide a rich web interface that combines hierarchical browsing of attributes and values with a solution for piecing together advanced queries. In addition, we provide a REST API for use by other software tools or for *ad hoc* scripting. Also, the TrackFind client tool in the GSuite HyperBrowser provides a helpful step-by-step search interface, where each selection limits the values that can be selected in the next step, in concordance with the metadata contents. In conclusion, we believe TrackFind and the FAIRtracks draft standard together will be able to greatly improve the findability of track files.

### Accessible

*A1. (Meta)data are retrievable by their identifier using a standardised communications protocol*

In our prototype implementation, indexed metadata are accessible through simple REST-based APIs that use the HTTP protocol, either from the Track Hub Registry ^xli^ or from TrackFind ^xlii^. The track data as such are available by FTP or HTTP, as hosted by the data provider. In some cases, *e.g*., for human variation data, access to the track data will be restricted, in which case we provide specific metadata fields for linking to the data usage policy and access control procedures. We also support the possibility to include identifiers to the raw files used to create the track, resolvable to external repositories. We advise to include such raw file references, as only referring to a higher-level dataset record will make it difficult to locate the exact files to use for researchers seeking to redo the upstream pipeline. However, in our test case of track files from the BLUEPRINT project, we were not (with reasonable effort) able to extract the EGA identifiers to the raw data files from the metadata available to us. Conversely, the ENCODE project provides access to raw files in an exemplary manner by providing multiple levels of references to original files, together with metadata about the pipeline that was used for each step.

*A2. Metadata are accessible, even when the data are no longer available*

Metadata persistence is a particularly important issue for genomic track files, as it is a type of data that is especially prone to becoming unavailable due to a technicality in how most genome browsers operate, in that they allow remote hosting of track files in file formats that support this, such as BigBed and BigWig ^28^. However, for this to work, the hosting web server needs to be configured with the ability for users to fetch only specific subparts of the files. Some common repositories for life science data do not support this, leaving the data providers with a need to host the files themselves, often achieved using temporary web hosting services. Thus, track data files often become unavailable after some years.

As a minimum, track metadata should persist. Even though Track Hub Registry (THR) and TrackFind could technically be able to fill such a role, we choose to depend on existing persistent repositories using DOI identifiers (e.g., Zenodo, see F1), as the operational model of THR allows submitters to delete their submissions, while the architecture of TrackFind is primarily designed around its search functionality. Zenodo provides storage connected to the CERN project infrastructure, to be maintained for at least 20 years ^xliii^.

One solution for providing persistent identifiers at the level of track files and track collections would be to implement a light-weight track registry service that builds on existing infrastructure. Such a service could: 1. Accept a DOI to a FAIRtracks-formatted document. 2. Generate and register globally unique identifiers for the contained track files and track collections. 3. Add these identifiers to the document. 4. Store the document in an existing persistent repository. 5. Allow fine-grained access to the metadata for each track or track collection. 6. Provide references (through at least Identifiers.org) to such fine-grained access to the document, as well as to the relevant records in TrackFind and elsewhere. 7. Possibly host web pages with track metadata using Bioschemas ^xliv^ mark-up for improved findability through standard web search engines. 8. Possibly support persistence of track data itself (e.g., through BioStudies ^xlv^), given the availability of the required storage space.

### Interoperable

*I1. (Meta)data use a formal, accessible, shared, and broadly applicable language for knowledge representation*

We tried to define the FAIRtracks model in a way that objects could be easily mapped to objects in other relevant metadata models ( Table 3). Thus, the FAIRtracks objects "Experiment", "Study", and "Sample" refer one-to-one to the INSDC counterparts with the same name and relationships ^xlvi^, with the exception that a FAIRtracks experiment might also refer to an *in silico* analysis, like a ChIP-seq peak calling run, in INSDC covered by the "Analysis" object. Compared to the ISA Abstract Model ^xlvii^, the FAIRtracks "Study" and "Sample" objects match identically named ISA objects, while the FAIRtracks "Experiment" object corresponds to either ISA "Assay" or "Process" objects. The addition of the "aggregated_from" attribute to the FAIRtracks "Experiment", allows downstream bioinformatics analyses to be traced stepwise back to the original laboratory experiment, in a more expressive way compared to the INSDC model, as well as in a simpler way compared to the ISA Abstract Model. The FAIRtracks "Track" object covers a single track file, which in INSDC is also confusingly covered by the "Analysis" object, and in the ISA Abstract Model by the "Data" object. We have also added an additional "raw_file_ids" attribute to the track record in order to allow matching track files with the exact original data files. Lastly, the FAIRtracks "Track collection" object is similar to the SRA "Submission", EGA "Dataset", Track Hub Registry "Track Hub", and ISA Abstract Model "Investigation" object. In addition, a track collection might also be used to describe *ad hoc* collections of track files extracted from different repository submissions, *e.g.*, to uniquely refer to a set of track files analysed in a research paper, thus providing a novel way to improve reproducibility of research findings. Used in this way, a FAIRtracks "Track collection" directly corresponds to the contents of the GSuite metadata file format, previously developed in context of the GSuite HyperBrowser ^1^, and we have thus also implemented tools to convert between the two formats.

**Table 3.** Mapping of FAIRtracks objects to objects in other metadata standards.

FAIRtrack	INSDC	ISA	Other	Comments
Track collection	SRA: Submission EGA: Dataset	Investigation	Track Hub Registry: Track Hub GSuite: Track collection	Can represent both original repository submissions, as well as other sets of track files, *e.g*., as analysed in a research paper.
Study	Study	Study		
Sample	Sample	Sample		
Experiment	Experiment & Analysis	Assay & Process		"aggregated_from" attribute allows provenance through all experimental steps
Track	Analysis	Data	Track Hub Registry/ GSuite: Track	"raw_file_ids" can link to original data files in case a full experiment trace is not available "source_coll_ref" links to the source track collection if current collection is an *ad hoc* mix

We have chosen JSON as the formal exchange format for track metadata. In addition to being the *de facto* standard for web APIs, JSON metadata can also be formalized at a meta-meta level using JSON Schema ^xlviii^. We thus developed the FAIRtracks draft standard as a set of JSON Schemas together with a validation service. Furthermore, we found the need to extend the FAIRtracks schemas with more property types than was allowed by the version of JSON Schema it follows (draft 7). Specifically, we extended validation to relationships between data (identifier existence and uniqueness within a FAIRtracks document, a type of validation that is outside the scope of JSON Schema ^xlix^), as well as the validation of ontology terms and Identifiers.org CURIEs. Our validation service is a generalization of software initially implemented in the context of the ELIXIR OpenEBench project ^l^, illustrating the general applicability of a possible standardized extended JSON schema and validation service for life sciences, as also investigated by the ELIXIR Implementation Study on Data Validation ^li^.

*I2. (Meta)data use vocabularies that follow FAIR principles*

FAIRtracks is following principle I2 by requiring that almost all text-based properties follow certain ontologies. For each such property, we have specified one or more supported ontologies, in most cases together with a set of ancestor terms. Each ontology term property consists of a pair of properties, one containing the term ID (as a URL), while the other contains the associated human readable label for the term. All of these relations are thoroughly validated by our FAIRtracks validation service.

We have not conducted thorough research of the FAIRness of the ontologies themselves, as this has been better handled by more dedicated studies ^lii^. We have, however, faced challenges in three areas: findability, provenance and cross-ontology references.

First, it is difficult to evaluate and compare the quality of ontologies for a particular domain. However, most relevant ontologies are helpfully registered in the Ontology Lookup Service ^liii^ (OLS) and the NCBO BioPortal ^liv^, which both provide support for ontology discovery based on a limited set of metadata fields. The OBO Foundry ^lv^ provides manually curated lists of OBO ontologies. FAIRsharing ^lvi^ annotates ontologies with richer metadata and provides a better experience for discovering ontologies. The AgroPortal ^29^ provides an expressive search interface built on a general model created by merging standards for metadata on ontologies, but the portal only supports ontologies in agronomy and related domains. In practice, we chose ontologies manually by getting an impression by searching for key terms, using *e.g.*, OLS or Zooma ^lvii^, followed by unstructured browsing of the resulting ontologies. Such a process was obviously highly subjective, in addition to having limited usefulness if one does not possess expert knowledge in the relevant domains. We would have been greatly helped in this if objective ontology metrics were readily available to guide us in our choices, *e.g.*, based on related databases, standards, and policies as annotated in FAIRsharing.

Secondly, the FAIR principle R1.2 recommends provenance of both data and metadata, however we are unaware of clear recommendations and solutions for capturing the exact version of an ontology that is used when annotating data. Changes in ontology structure and content may create problems for automatic validation or search. Initially, we figured that a specific version of our FAIRtracks draft standard should require ontology terms according to specific versions of each ontology, ensuring consistent validation of ontology terms. However, this would require us to release an update of FAIRtracks for every new release of a supported ontology, and it would also complicate the implementation of the validator. In the end, we opted to not require specific versions of the ontologies and instead validate against the latest version of each. However, we require that the ontology versions are captured for each metadata document. With this solution, ontology changes might break validation of previously validated metadata records, but since provenance is available, one should at least be able to resolve any issues.

Thirdly, managing cross-ontology references is a fundamental challenge that was not fully explored within the scope of this study. We therefore in most cases chose to support only a single ontology for each term. Without this limitation, the list of allowed values for a field would typically end up containing variations of the same terms, making downstream use unnecessarily cumbersome, *e.g.,* when selecting filters for a query. On the other hand, limiting to only one ontology for each field places a burden on the metadata providers and curators, as one would probably not be able to find an ontology scheme that suits the particular use case or domain for every track collection. FAIRtracks can potentially allow non-redundant merging of several domain-focused ontologies by making heavy use of ancestor term restrictions. Regardless, there is a clear need for powerful tools to translate terms across ontologies.

For our BLUEPRINT test case, we made good use of the Ontology Xref Service (OXO) ^lviii^ to transform existing ontology terms in order to comply with our FAIRtracks draft standard. OXO is still under development and the conversion process required some manual oversight, for example to select between alternative mappings. We note that some projects such as GWAS Catalog ^lix^ already automated the process of batch transformation and mapping of structured metadata, tailored to their use cases (pers. comm. H. Parkinson).

The FAIRtracks draft standard includes two novel fields in the Track object that we believe will prove to be very useful for downstream users, but that we were unable to properly populate using existing ontologies. We thus decided, for now, to provide limited vocabularies for them in the JSON schema itself. The aim of both fields is to provide a simple theoretical framework to categorize track files according to their potential usage. Track data, by definition, is formed downstream of some data condensation process, typically a pipeline of software tools that simplifies a set of raw sequence reads of varying quality into a condensed track file with relatively high-confidence data. Several existing ontologies contain terms describing the experimental technique, *e.g*., "ChIP-seq assay" ^lx^, as well as the pipelines, *e.g.*, "Peak calling" ^lxi^, but there are very few terms available to describe the results of those pipelines, that is, specific types of condensed data represented as track files, *e.g.*, "Broad peaks". In contrast to file formats, *e.g*., "BigBed" ^lxii^, our first novel field "type_of_condensed_data" describes the condensed data itself, and thus its interpretation, rather than its representation. The second field, "genomic_track_type", builds upon a previous study ^7^ delineating tracks according to their basic geometric properties when viewed as mathematical objects along a one-dimensional line ( *i.e.* the coordinate system defined by the reference genome assembly). Our initial vocabularies ^lxiii^ are not meant to be comprehensive lists, but rather starting points of a process leading to the inclusion of such terms into a relevant ontology, possibly the EDAM ontology ^lxiv^ where such data-describing terms might fit well. 

As the FAIRtracks draft standard is developed primarily as a proof-of-concept and a starting point for a common metadata standard, our initial choices of supported ontologies are suggestions open for debate, mostly based upon our opinions and that of early adopters. However, as the standard matures, we expect the choices to converge towards a set of broadly supported, and FAIR, ontologies.

*I3. (Meta)data include qualified references to other (meta)data*

Rather than aiming for metadata completeness, FAIRtracks aims to improve track data and metadata availability and reuse by bridging the current gap between track data repositories on one hand, and analysis scripts, tools and frameworks on the other. As discussed in detail above (F2, F3), FAIRtracks thus supports and recommends the inclusion of global identifiers to external records containing more detailed metadata. We require that such global identifiers are represented in CURIE form to be resolvable through the Identifiers.org ^lxv^ service, one of the ELIXIR Recommended Interoperability Resources ^lxvi^. Due to a recent technical collaboration ^25^, all supported CURIEs should also be resolvable through the US-based N2T.net service ^lxvii^, but this has not been tested. A mapping service in the opposite direction to Identifiers.org, *i.e.*, from existing URIs to the corresponding CURIEs, would be highly useful for adapting existing metadata to the FAIRtracks draft standard.

Historically, lacking or inaccurate identification of the genome assembly version of track files has been a source of great confusion ^6^, and can serve as an example. Even when assembly identifiers such as "hg19" and "GRCh37" are included in the track metadata, they lack the required context for interpretation, causing researchers to have to delve into the intricacies surrounding naming conventions for human genome assemblies. Full URIs, such as " https://www.ncbi.nlm.nih.gov/assembly/GCF_000001405.13", refer uniquely to unambiguous records, and thus improve on simple string and/or number combinations. However, using URIs require the records to persistently be available at a particular location. The CURIE counterpart "insdc.gca:GCF_000001405.13" is location-independent, and should resolve ^lxviii^ into both the above URI and a URI for a record stored within the EMBL/EBI infrastructure.

As a side note, using such a CURIE does not solve the inconsistencies on chromosome naming conventions for certain species ( *e.g.*, human and mouse). Even though the Genome Reference Consortium (GRC) standard denotes chromosomes using simple numbers and capital letters ( *e.g.*, 1, 2, X, ...), the historical convention of the UCSC Genome Browser ( *e.g*., chr1, chr2, chrX, ...) is still the *de facto* standard for most track files. In FAIRtracks, we have tried to solve this issue by requiring an additional field "annotation_name" containing either the standard name of the genome assembly ( *e.g.*, "GRCh37") or one of the registered synonyms ( *e.g.*, "hg19"), according to the chromosome naming scheme used in the track file. Ongoing GA4GH-supported community efforts to standardize unique identifiers for collections of sequences should improve the situation, and will likely be adopted in a later version of FAIRtracks.

A potential limitation to our extensive use of resolvable identifiers is the added requirement that all identifiers are openly resolvable from a web endpoint. In the case of the BLUEPRINT test metadata, we discovered for instance that the EGAX experiment identifiers were not resolvable (only EGA dataset and study types were supported ^lxix^). Adding such support would require the implementation of a dedicated API endpoint, a decision that in many cases is not up to the metadata provider. The current consequence is that our test BLUEPRINT metadata set does not validate correctly. It remains to be seen whether increased usage of CURIEs over time will decrease the occurrence and impact of such problems.

### Reusable

*R1. Meta(data) are richly described with a plurality of accurate and relevant attributes*

FAIR principle R1 overlaps somewhat with principle F2, but while F2 focuses on discovery of the dataset, R1 focuses on the user's ability to decide whether the data is useful in their particular research scenario. We believe our minimal set of required metadata fields, extracted from existing standards and selected based on years of experience in track file analysis, provides a breadth of generally relevant fields for most typical usage scenarios. Also, FAIRtracks enforces quite stringent limitations on the contents of the fields in order to provide sufficient accuracy. Furthermore, richer metadata can be added to external resources referred to by the FAIRtracks records, or such metadata can be added directly to the FAIRtracks records themselves. 

We realized during the implementation process that two seemingly conflicting views of the purpose of our draft standard were held within our project group. These two views can be summarized as follows:

#1. Facilitating the minimal set of fields that a submitter of a track collection would need to fill out in order to get the metadata approved as FAIR
#2. Facilitating the minimal structured metadata that is directly useful for downstream usage

As an example, consider the case of ontology terms. Metadata submitters should only need to provide the correct identifier for each term. However, those identifiers are incomprehensible for downstream human users. If the standard were to follow view #1, all downstream tools would thus need to implement ontology lookup functionality, making the process unnecessarily cumbersome for researchers, especially in simple scripts. However, if the standard were to fully follow idea #2, the burden of ontology lookup would be placed on the data providers. Similar conflicts were present for other issues. Fortunately, we discovered a technique that would help us adequately meet the requirements of both usability and ease of metadata entry: We implemented an augmentation service ^lxx, lxxi^ that, based on the minimal fields required by the FAIRtracks JSON schema (supporting view #1), automatically generates an extra set of "augmented" fields, containing human-readable or otherwise useful content for downstream users, removing the need for ontology lookup and similar (supporting view #2).

We have also found that augmentation can be very useful for integrating heterogeneous datasets, *e.g.*, for multi-omics analysis. Rich and accurate metadata fields are often highly specific to particularities of a type of datasets, *e.g.*, related to sampling process or experimental technique. However, having to gather and integrate metadata values from such specialized fields is impractical if one is integrating a number of different dataset types. Consider, *e.g.*, two samples: one is derived from an established cell culture, while the other was created by isolating a particular cell type from a biological sample. In the first case, a main categorization of the sample could be represented by a "cell line" field, which would only accept values according to an ontology of cell lines, *e.g.*, "H1-hESC". In the other case a controlled "cell type" field could be used for the categorization, *e.g.*, as "B cell, CD19 positive". However, a researcher might want to merge such fields into one for a broad scan of available data. Or similarly, a researcher might want to combine ChIP-set tracks of transcription factor binding alongside histone modification experiments, or even GWAS variants for different phenotypes. The 2-standards-in-1 design of FAIRtracks allows the support for both stringent validation of clearly defined fields to ease metadata entry, while at the same time providing merged fields with harmonized content. The FAIRtracks draft standard includes two such summary fields populated by the augmentation service. First, in the "Sample" object, the "sample_type → summary" field summarizes the other child fields of "sample_type", *i.e*. "abnormal_cell_type", "cell_line", "cell_type", and "organism_part", according to rules determined by the value in the field "biospecimen_class". Second, in the "Experiment" object, the field "target → summary" is an augmented field containing the summary of the other child fields of "target", *i.e.* "gene_id", "gene_product_type", "macromolecular_structure", "phenotype", "sequence_feature", and "target_details", according to rules determined by the value of the field "technique". In the case of, *e.g.*, ChIP-seq transcription factor (TF) experiments, the HUGO Gene Nomenclature Committee (HGNC) identifier for the TF should be entered in the "gene_id" field, with any post-translational modifications in the "target_details" field. Such rules are enforced by heavy use of JSON Schema validation structures.

It can be argued that it is risky to define a general standard to include metadata requirements based on certain particularities of specific experimental types and sample types, as the maintenance of up-to-date and domain-relevant logic might become difficult. However, we believe the advantages of such limited logic significantly outweighs the extra burden. We envision to gradually extend and improve the logic and structures with a group of early adopters based on real-world metadata.

*R1.1. (Meta)data are released with a clear and accessible data usage license*

The FAIRtracks draft standard annotates track data files with a combination of the machine-readable fields using the GA4GH-approved Data Use Ontology ^lxxii^, as well as a URL field linking to human-readable data use license/policy documents.

*R1.2. (Meta)data are associated with detailed provenance*

It has been paramount to the design of the FAIRtracks draft standard, as well as to the supporting infrastructure, to allow tracing the provenance of track data and metadata in a transparent manner. This is handled at many levels:

1. FAIRtracks is an intermediate metadata standard designed to include references to external metadata records, including the original records from which the FAIRtracks metadata has been derived. Those records will often contain additional provenance information.
2. As detailed under I1 above, FAIRtracks supports a mechanism for tracing the data stepwise back through various bioinformatics analyses and laboratory experiments, by using the field "aggregated_from" in the "Experiment" object. One can attach external records to every step, if available.
3. A separate mechanism, the field "raw_file_ids" in the "Track" object, can be used to at least refer to the original data files, even if a stepwise history is not available.
4. The "Experiment" object contains the free-text fields "lab_protocol_description" and "compute_protocol_description" to record information about the experiment setup, either directly or as a URI to an external record. Optimally, such provenance information should be stored in a structured manner, according to community standards for provenance information, such as Provenance Notation (PROV-N) ^lxxiii^ or CWLProv ^30^. Records containing provenance information could be attached to globally unique and persistent identifiers and made available ( *e.g.*, as RO-Crate ^lxxiv^) ^31^, possibly also in a searchable registry, such as the ongoing development of WorkflowHub ^lxxv^. As research and implementation of experiment provenance metadata is out of scope of the current project, we settled for free-text fields.
5. In the top-level metadata relating to a FAIRtracks document itself, we include fields for version information and date, as well as "derived_from" and “augmented_from” fields that can be used to trace the evolution of the metadata itself. Such provenance tracing requires globally unique and persistent identifiers for track metadata content, as we argue for in section F1 above.

*R1.3. (Meta)data meet domain-relevant community standards*

Currently, efforts of FAIRifying genomic track data are mostly restricted to a few high-volume providers, with related metadata guidelines. Those few community standards that exist are either defined for specific domains, *e.g.*, human epigenomics (IHEC) or animal genomes (FAANG), or in relation to specific tools or services, *e.g.*, the TrackHub format specification or the Track Hub Registry requirements. None of the few community standards have been designed for general use and in explicitly in accordance with the FAIR principles. We have designed the FAIRtracks draft standard to combine broadness of metadata content with enough stringency to be trusted by downstream users and tools, based on years of experience as consumers of genomic tracks. We thus aim for the FAIRtracks draft standard to evolve into such a community standard, helped by the establishment of useful services that facilitate FAIR sharing of genomic track files, not least through the TrackFind web server and search API. We aim to approach core providers of data and tools to build an initial catalogue of content and integrations. As soon as a sizable amount of track metadata is available, we expect the momentum to integrate with TrackFind to increase, propelling further metadata FAIRification. For further adoption from data providers, we plan to provide automatic certification by flagging Track Hub Registry submissions that is verified to contain FAIR metadata, and we recommend the implementation of a track metadata registry. Such a registry should require submissions according to a FAIR community standard, possibly evolved from the FAIRtracks draft standard. We also intend to make FAIRtracks-related contributions to other FAIR sharing efforts, such as to the FAIRplus Cookbook, which is a resource that collates FAIRification recipes primarily in connection to the Innovative Medicines Initiative ^lxxvi^, but also with an aim to reach as wide an audience as possible ^32^.

Furthermore, to simplify metadata transformation and curation, we envision taking part in community efforts to implement services for computer-assisted FAIRification of metadata, including mechanisms for curation and transformation of features, both on a per-record basis and in batch. It would be imperative for such services to provide advanced support for curating ontology terms, including guided ontology matching, support for generating CURIE identifiers, and integration with the FAIR metadata validation service.

For a community standard to evolve, the consolidation of a relevant community needs first to take place. The current project group, though relatively small, has included members with solid experience from tools development (GSuite HyperBrowser, EPICO); from track data and metadata production (BLUEPRINT, FANTOM); from consortia-based management of track repositories and related standards (IHEC, ICGC); as well as from the registration and dissemination of user-submitted track collections (The Track Hub Registry). An obvious weakness of our approach is the uncertain outlook on community acceptance of the FAIRtracks draft standard. Typically, standards are created either as part of early, domain-specifying implementations, or by larger field-dominating groups. However, standards also sometimes arise if they fill specific gaps of general importance, and it is our hope that a broader community will arise downstream of our efforts. The current study represents the initial step in this process.

## Conclusion

We believe that with the proposed FAIRtracks draft standard and accompanying service infrastructure, we have taken a useful step towards realizing the underexploited value of genomic track datasets. However, whether this effort will realize the full potential will ultimately be decided by the scientific community, to the extent that the standard will be adopted and implemented.

## Data availability

No data is associated with this article.

## Software availability

FAIRtracks standard (including FAIRified BLUEPRINT metadata): https://github.com/fairtracks/fairtracks_standard (License: CC-BY 4.0)

Track Hub Registry: https://github.com/Ensembl/trackhub-registry (License: Apache 2.0)

FAIRtracks validator (License: LGPL 2.1) and FAIRtracks validator server (License: AGPL 3): https://github.com/fairtracks/fairtracks_validator

FAIRtracks augment service: https://github.com/fairtracks/fairtracks_augment (License: Apache 2.0)

FAIRtracks format conversion service: https://github.com/fairtracks/fairtracks_json_to_gsuite (License: MIT)

TrackFind: https://github.com/elixir-no-nels/trackfind (License: MIT)
