# Supplementary material for: Recommendations for the FAIRification of genomic track metadata
Source: F1000Res. 2021 Apr 1;10:ELIXIR-268. [Version 1] doi: 10.12688/f1000research.28449.1 (PMC8226415; doi:10.12688/f1000research.28449.1)
Supplement: Peer review history

# Peer review: Reviewer response for version 1

**DOI:** 10.5256/f1000research.31488.r87124

**Published:** 2021-06-24

**Authors:** Granger Sutton

**Affiliations:** 1 J. Craig Venter Institute, Rockville, MD, USA

This is a well written opinion article describing a proposed set of standards for genomic track metadata. As the authors point out in the conclusion this is merely a first step in the process towards community acceptance but without draft proposals such as this there is nothing to work towards. I am not an expert on many aspects of the proposed standards but am certainly familiar with many of the issues and use cases. This seems like a reasonable effort at starting the discussion. The proposal is enhanced by the prototype use of the standard and the availability of tools for using it.

 I had two minor quibbles with the article. First, it is not clear to me that “A survey of metadata keys available on the track hubs suggests a great disparity in usage as provided in Table 1.” is correct. Earlier in the article Table 1 was described to contain “Domain-specific metadata standards exist that guide current efforts to annotate genomic datasets (see Table 1 for a summary of object types across these data models and their required attributes).” which does appear to be correct. Perhaps both are correct, but this needs to be explained if so. I think a better description of what is in Table 1 needs to occur earlier in the article.

 Second, “This testing phase clarified a need for an automated intermediate step between metadata curation and consumption, which in most cases simply meant to augment resolvable identifiers with human readable versions of the same.” needs to be clarified perhaps with an example.

Is the topic of the opinion article discussed accurately in the context of the current literature?

Yes

Are arguments sufficiently supported by evidence from the published literature?

Yes

Are all factual statements correct and adequately supported by citations?

Yes

Are the conclusions drawn balanced and justified on the basis of the presented arguments?

Yes

Reviewer Expertise:

pan-genome analysis, whole genome shotgun assembly, genome annotation, homology search, multiple sequence alignment

I confirm that I have read this submission and believe that I have an appropriate level of expertise to confirm that it is of an acceptable scientific standard.

# Peer review: Reviewer response for version 1

**DOI:** 10.5256/f1000research.31488.r82701

**Published:** 2021-05-21

**Authors:** Max Haeussler

**Affiliations:** 1 Department of Biomolecular Engineering, University of California Santa Cruz, Santa Cruz, CA, USA

This article proposes a standard for adding metadata to track hubs. The "track hub" file format was defined by the UCSC Genome Browser group to be able to display genome annotations from static files, without having to setup a server like DAS. It was not meant to be a general data exchange format, but nevertheless includes various ways to add metadata to the tracks. But the metadata key names are not defined and there are no required keys. This makes it hard to find annotation tracks, as in practice, most track hubs do not use the metadata at all, so all one is left with are the short and long human-readable labels. This makes finding and automated analysis of tracks (e.g. Chip-seq, variants, CNVs) harder than necessary.

 To solve this problem, the article proposes a system where a JSON file is added to the track hub with data on study/sample/experiment and tracks and a given set of recommended key/value items for each track specified via ontologies.

 The proposed solution sounds reasonable. If adopted by everyone, it would solve make track hubs adhere to the FAIR principles much better than at the moment. Similar to the authors themselves, I have doubts that this will be adopted quickly, but the paper is still sound.

 The paper is well written as it is, I have no minor comments, just a few general ones:

 0) You say that the format must be "clear and useful". (Does this mean that simplicity was not a design goal?) I think the article would be easier to understand if you show a simple JSON example. The data format is explained, but examples go a long way. I know that you link an example on your Github page, but in an article where you discuss a new data format, an example right in the article of an example allows the reader to check that the format is indeed clear and useful. 

 1) You note that the UCSC track hub specification already includes three ways to define metadata. You note that hardly any track hub uses the existing very minimal metadata system. Do you think that yet another metadata format will be used more than the current one? If yes, why? Do you have suggestions what be done by genome browsers, journal editors or databases to encourage hub developers to add more metadata, e.g. when ranking search results, showing track hubs, or when requiring authors to provide track hubs, like NAR does? These could fit very well in the discussion at the end.

 2) The existing metadata formats are either very simple (inline or tsv) or very compact (tagStorm). tagStorm and inline are human-readable text files, they can be typed easily in a text editor and they support, for example, comments. JSON does not support comments and is not as easy to type by humans. Do you think that is a disadvantage of JSON?

 3) "The Track Hub Registry is a global centralised collection of public track hubs" - the EBI's track hub registry is copying the UCSC public hubs list, which preceded it. The UCSC public hubs list is a curated list of track hubs submitted to UCSC and manually quality-checked. The Track Hub Registry adds to this list thousands of track hubs auto-generated from ENA submissions. In addition, some track hubs may have been submitted by users through the track hub registry API, but I see no way to find out which ones were added in which way. I think you could mention the UCSC public hubs list as a global, centralized and curated collection of public track hubs with certain quality standards.

 "The Track Hub Registry ^xxix^ allows data producers to register and share trackhub files. These files are then parsed and indexed by the Ensembl and UCSC genomic browsers" - There is a confusion in the article between the track hub registry and the UCSC Genome Browser. The Genome Browser does not "index" the track hub registry. By clicking the link on the registry, you open this hub. The UCSC Genome Browser indexes its own public hubs list for searching, so when you search for tracks, the Genome Browser will bring up tracks from the public hubs list, but not from the registry.

 4) You structure your data model around the usual (study, sample, experiments) entities, similar to the SRA. What happens if a dataset has a hierarchical structure within the samples? As you know, this is extremely common outside of large scale projects like ENCODE and Blueprint: A biologist starts with three cell cultures, runs RNA-seq, knocks out a gene in them, does RNA-seq, treats with some drug and does Chip-Seq on the gene-knockout-cell culture. In your data model, these samples would all be independent, and the relationships between them, the tree structure, are lost. To me it seems that your data model doesn't support inheritance or relationships between samples or between experiments, is this correct?

Is the topic of the opinion article discussed accurately in the context of the current literature?

Yes

Are arguments sufficiently supported by evidence from the published literature?

Yes

Are all factual statements correct and adequately supported by citations?

Yes

Are the conclusions drawn balanced and justified on the basis of the presented arguments?

Yes

Reviewer Expertise:

genome browsers, genome annotations

I confirm that I have read this submission and believe that I have an appropriate level of expertise to confirm that it is of an acceptable scientific standard.
